# Tumor Immune Microenvironment Characterization Identifies Prognosis and Immunotherapy-Related Gene Signatures in Melanoma

**DOI:** 10.3389/fimmu.2021.663495

**Published:** 2021-05-06

**Authors:** Dan Liu, Xue Yang, Xiongzhi Wu

**Affiliations:** ^1^ Tianjin Medical University Cancer Institute and Hospital, National Clinical Research Center for Cancer, Tianjin's Clinical Research Center for Cancer, Key Laboratory of Cancer Prevention and Therapy, Key Laboratory of Cancer Immunology and Biotherapy, National Human Genetic Resources Sharing Service Platform, Tianjin, China; ^2^ Department of Medical Oncology, Tianjin Medical University General Hospital, Tianjin, China

**Keywords:** melanoma, tumor microenvironment, immune cell infiltration, prognosis, immune therapy

## Abstract

**Background:**

The tumor microenvironment (TME) involves infiltration of multiple immune cell subsets, which could influence the prognosis and clinical characteristics. The increasing evidence on the role of tumor-infiltrating lymphocytes (TILs) in primary and metastatic melanomas supports that the immune system is involved in the progression and outcomes of melanoma. However, the immune infiltration landscape in melanoma has not been systematically elucidated.

**Methods:**

In this study, we used CIBERSORT and ESTIMATE algorithms to analyze immune infiltration pattern of 993 melanoma samples. Then we screened differential expression genes (DEGs) related to immune subtypes and survival. The immune cell infiltration (ICI) score was constructed by using principal-component analysis (PCA) based on immune signature genes from DGEs. Gene set enrichment analysis (GSEA) was applied to explore high and low ICI score related pathways. Finally, the predictive ability of ICI score was evaluated in survival prognosis and immunotherapy benefit.

**Result:**

We identified three ICI clusters and three gene clusters associated with different immune subtypes and survival outcomes. Then the ICI score was constructed, and we found that high ICI score exhibited activated immune characteristics and better prognosis. High ICI score was significantly enriched in immune pathways and highly expressed immune signature genes. More importantly, we confirmed that melanoma patients with high ICI score had longer overall survival and rate of response to immunotherapy.

**Conclusion:**

We presented a comprehensive immune infiltration landscape in melanoma. Our results will facilitate understanding of the melanoma tumor microenvironment and provide a new immune therapy strategy.

## Introduction

Malignant melanoma is one of the most severe skin cancers, with a higher risk of metastasis and mortality rates. Approximately 324,635 incident cases and 57,043 related deaths were reported worldwide in 2020 (1). In the past decades, immune checkpoint inhibitor therapy targeting cytotoxic T-lymphocyte antigen 4 (CTLA-4), programmed cell death receptor 1 (PD-1), and programmed cell death ligand 1 (PD-L1) have resulted in impressive outcomes in patients with malignant melanoma (2). However, approximately 40–65% of melanoma patients have no response to or develop relapse after anti-PD-1 therapy due to primary or acquired resistance, and over 70% of patients experience anti-CTLA-4 treatment failure (3).

Currently, PD-L1 expression, tumor mutation burden (TMB), and microsatellite instability-high (MSI-H) are the primary biomarkers for guiding the clinical practice of immune therapy and predicting survival benefit in several types of cancer (4, 5). However, neither PD-L1 expression nor TMB is a perfect biomarker for immune therapy prediction in melanoma, as responses are also observed in PD-L1-negative and low TMB patients. Despite efforts to identify new biomarkers predictive of the benefits of immune therapy, such as ARID2 and tumor MHC-I expression (6, 7), no robust biomarker has been established to drive clinical practice. Therefore, identification and characterization of potential biomarkers and their application in combination with immune therapy are urgently needed.

The tumor microenvironment (TME) involves infiltration of multiple immune cell subsets, and the pattern of immune cell infiltration (ICI) could influence the prognosis and clinical benefit of melanoma immune therapy (8, 9). TME is a vital factor that determines the efficacy of anti-tumor immune therapy (10). Studies have reported that acral and mucosal melanoma patients with a lower number of total tumor-infiltrating lymphocytes (TILs) have a poorer response to immune checkpoint inhibitors than does their cutaneous melanoma counterparts (11, 12). Primary and acquired resistance to immune checkpoint blockade commonly occurs due to a tumor immune escape mechanism regulated by the TME (13). One study proposed the degree of lymphocyte infiltration as an independent prognostic factor of disease-free survival (DFS) in melanoma patients, with a higher TIL grade associated with a lower risk of death (14). The characteristics of ICI can serve as an effective prognostic biomarker and predictive indicator of response to immune therapy (15). Therefore, it is necessary to gain a comprehensive understanding of the immune infiltrate characteristics in melanoma.

This study aimed to characterize the ICI landscape of melanoma. Toward this goal, we used two algorithms, namely, CIBERSORT and ESTIMATE, and further constructed ICI scores that can be used to predict survival prognosis and immune therapy benefit, based on the immune infiltration pattern and immune signature genes.

## Materials and Methods

### Melanoma Datasets and Processing

Melanoma microarray datasets were searched and downloaded from the Cancer Genome Atlas (TCGA) (https://portal.gdc.cancer.gov/) and the Gene Expression Omnibus (GEO) (https://www.ncbi.nlm.nih.gov/geo/). The inclusion criteria were as follows: 1) human malignant melanoma sample, except mucosal and uveal melanomas; 2) mRNA expression profiling by microarray; 3) sample size ≥40; and 4) complete clinical information including overall survival time and state. After excluding repetitive samples, the following six datasets that included 993 melanoma samples were analyzed: GSE65904, GSE59455, GSE54467, GSE465517, GSE19234, and TCGA-SKCM cohorts. Detailed information on the microarray datasets is shown in **Table S1**.

The raw data from the GEO database was performed normalized including quality control, background correction, logarithmic conversion and remove batch effects, then the gene probes were annotated. The RNA-sequencing data (FPKM values) downloaded from the TCGA database were transformed into transcripts per kilobase million values, which were more similar to the microarray results facilitated to analysis.

### Consensus Clustering of Tumor-Infiltrating Immune Cells

The levels of ICI for melanoma were quantified using the CIBERSORT algorithm and LM22 gene signature. CIBERSORT is a deconvolution algorithm that uses a set of reference gene expression values (a signature with 547 genes) to evaluate cell type proportions in data from bulk tumor samples with mixed cell types (16). The LM22 gene signature could discriminate 22 human immune cell phenotypes, including B cells, T cells, natural killer (NK) cells, macrophages, dendritic cells (DCs), and myeloid subsets (16). The immune and stromal scores of each melanoma sample were calculated using the ESTIMATE method (17). Unsupervised clustering was carried out to identify patterns of ICI. The analysis was performed R package “ConsensuClusterPlus,” and iteration was 1,000 times to ensure classification stability.

### Identification of Differentially Expressed Genes and Generation of ICI Gene Signatures

Based on the clustering results of ICI, all samples were grouped into three clusters to identify differentially expressed genes (DEGs) associated with the immune subtypes. The analysis was performed using the R package “limma”, and the threshold value was an absolute fold-change >1 and an adjusted P value of <0.05.

The unsupervised clustering method was performed to group samples according to the DEG values. The DEGs that were positively and negatively correlated to the clusters were defined as the ICI signature genes A and B, respectively. Signature genes A and B have differential expressions in different gene clusters, and differential expression pattern also exits between signature genes A and B in one gene cluster.

The Boruta algorithm was applied to reduce the dimensions of the ICI gene signatures A and B. We applied principal component analysis (PCA) to construct the ICI score. The algorithm chooses best feature genes sets by a feature selection and extraction method, which has proven better statistical power. Without loss of generality, the first principal component (PC1) of matrix is selected for analysis as a signature score (18). We performed PCA to score signature genes A or B for each sample, and got two PC1 values for each sample, as PC1A and PC1B. We then used a method similar to the Gene expression grade index (19) to define each sample ICI score:

ICIscore= ΣPC1A −ΣPC1B

### Functional Enrichment Analysis

ICI signature genes A and B were subjected to Gene Ontology (GO) enrichment analysis. All the samples were subjected to gene set enrichment analysis (GSEA) and were divided into the ICI high score group and the ICI low score group. The GSEA software was downloaded from the GSEA website (http://software.broadinstitute.org/gsea/index.jsp). Enrichment results were visualized using the R package “ggplot2”.

### Somatic Alteration Data Download and Analysis

The melanoma mutation data were downloaded from the TCGA database (https://www.cancer.gov/tcga/). The raw data were extracted and also grouped by high and low ICI scores. We evaluated the total number of non-synonymous mutations in all melanoma samples, and the somatic alterations in melanoma driver genes in the high and low ICI score groups. The R package “maftool” was used to identify driver genes.

### ICI Scores and Clinical Characteristics Immune Therapy Benefit

Data from three immune therapy cohorts, that is, GSE78220, CA209308 and GSE19423 were downloaded to validate the value of the ICI score for predicting the immune therapy benefit. From the GSE78220 cohort, 27 melanoma samples treated with the anti-PD1 drug pembrolizumab were included. From the CA209038 cohort, which was downloaded from https://github.com/riazn/bms038_analysis, 68 melanoma samples treated with the anti-PD1 drug nivolumab were included. After excluding repetition or samples without clinical information, 50 samples were included in the analysis. From the GSE19423 cohort, 48 primary bladder cancer samples treated with Bacillus Calmette–Guérin (BCG) immunotherapy were included. A metastasis melanoma cohort GSE22154 was downloaded to validate the predictive value of the ICI score in metastasis, which included 22 metastatic melanoma samples.

### Statistical Analyses

Between-group comparisons were performed using the Wilcoxon test, while comparisons among more than two groups were conducted using the Kruskal-Wallis test. Survival curves were generated using the Kaplan-Meier method and compared using the log-rank test. The R package “survminer” was used to evaluate the best cutoff values of dataset based on the relation between overall survival and ICI score. The heatmap was constructed using the R package “pheatmap.” The correlation between the ICI score group and somatic mutation frequency was analyzed using the chi-square test, and the correlation coefficient was evaluated with Spearman analysis. All statistical analyses were performed using R (version 4.0.3) or GraphPad Prism (version 6.01), and two-tailed P<0.05 was considered statistically significant

## Results

### Landscape of Immune Cells Infiltration

**Figure 1:**
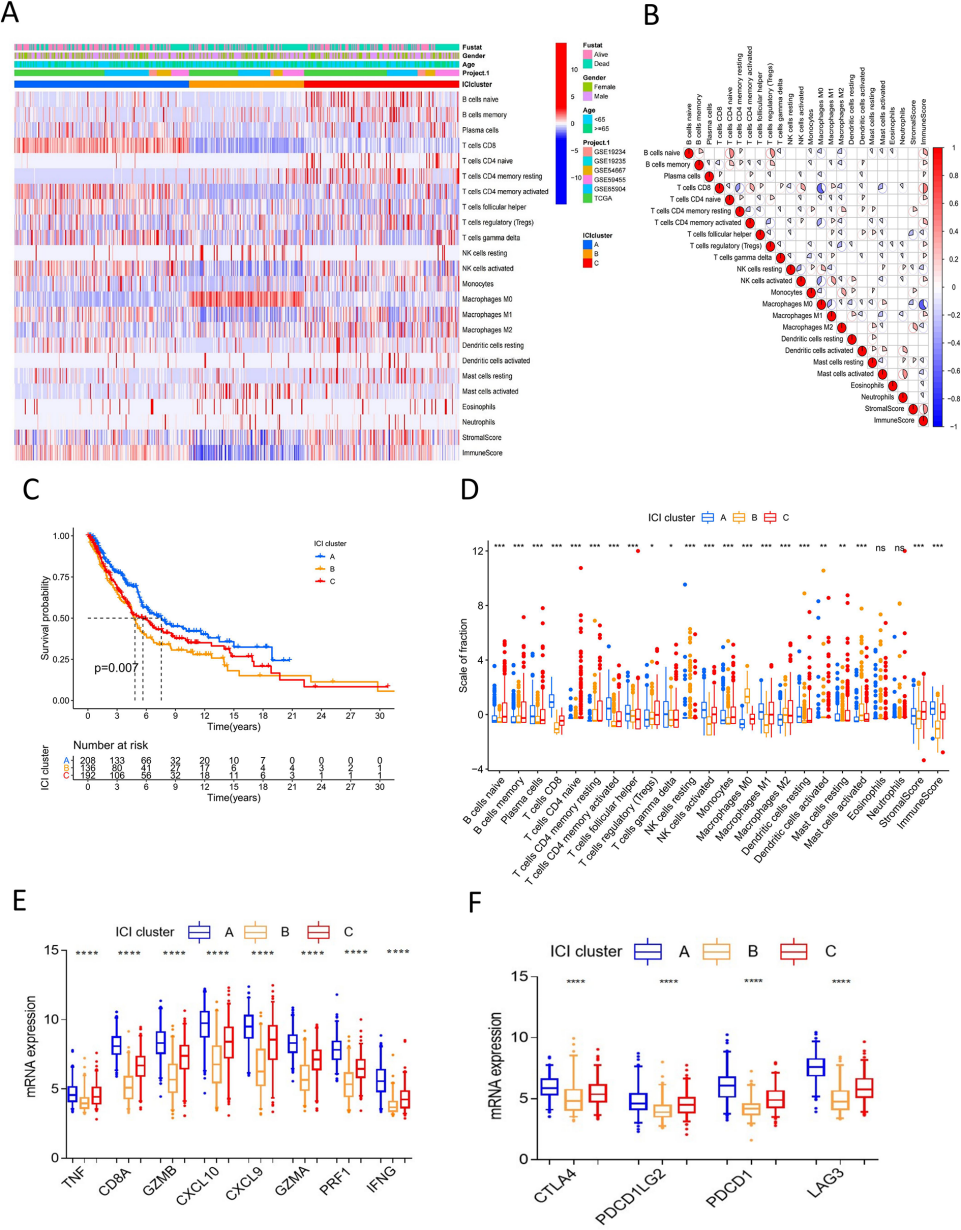
Landscape of immune cells infiltration in melanoma. **(A)** Unsupervised clustering heatmap of immune cells infiltration for all melanoma samples. Rows represent tumor-infiltrating immune cells, and columns represent samples. **(B)** The correlation coefficient heatmap of immune cell interaction. **(C)** Kaplan-Meier curves of overall survival for ICI cluster A–C. Log rank test P = 0.007. **(D)** The box plot of immune cells fraction in ICI cluster A–C. *P<0.05; **P < 0.01; ***P<0.001; ****P<0.0001; ns: no significance. **(E)** The box plot of immune activity related signature genes expression (CXCL9, CXCL10, TNF, IFNG, CD8A, GZMA, GZMB, PRF1) between ICI cluster A–C, ****P<0.0001. **(F)** The box plot of immune checkpoint signature genes expression (CTLA4, PDCD1, PDCD1LG2, LAG3) between ICI cluster A-C, ****P<0.0001.

We searched public databases and screened microarray datasets, six melanoma microarray datasets including 993 melanoma samples were brought into analysis. Mucosal and uveal melanomas samples were excluded for significant difference from cutaneous melanoma in presentation, genetic profile, staging, response to treatment and patterns of progression (20, 21). Detailed information of the included datasets is shown in **Table S1**. We employed the CIBERSORT and ESTIMATE algorithms to calculate the levels of ICI, immune score, and stromal score of each melanoma sample (**Table S2**). The ICI pattern was analyzed using unsupervised clustering. The optimal cluster pattern was three immune subtypes, that is, ICI clusters A-C (**Figures S1B–E**). The ICI heatmap showed that the immune cells were composed of different immune clusters (**Figure 1A**). The correlation coefficient heatmap showed the landscape of immune cell interaction in melanoma TME (**Figure 1B**). Cluster A was characterized by a high immune score with increased infiltration of CD8 T cells, activated NK cells, CD4 T memory activated cells, and T follicular helper cells. Cluster B exhibited low infiltration levels for most immune cells, but with a high density of M0 macrophages, activated mast cells, and resting NK cells. ICI cluster B had minimum immune score and stromal score. ICI cluster C showed high infiltration of naive B cells, memory B cells, Tregs, M1 macrophages, and M2 macrophages.

Survival analysis to explore the prognostic value of the ICI clusters showed that ICI cluster A had better survival outcomes. Meanwhile, ICI cluster B was associated with a worse prognosis. Cluster C was characterized by an intermediate overall survival (log-rank test P=0.007, **Figure 1C**). A consistent result was observed in the boxplot of immune cell fraction for the three ICI clusters, and this was further confirmed with the Kruskal-Wallis test (**Figure 1D**). The expression of immune activity-related signature genes (CXCL9, CXCL10, TNF, IFNG, CD8A, GZMA, GZMB, and PRF1) and immune checkpoint signature genes (CTLA4, PDCD1, PDCD1LG2, and LAG3) were the highest in ICI cluster A and the lowest in ICI cluster B (P<0.0001, **Figures 1E, F**).

### Identified Immune Gene Subtype

**Figure 2:**
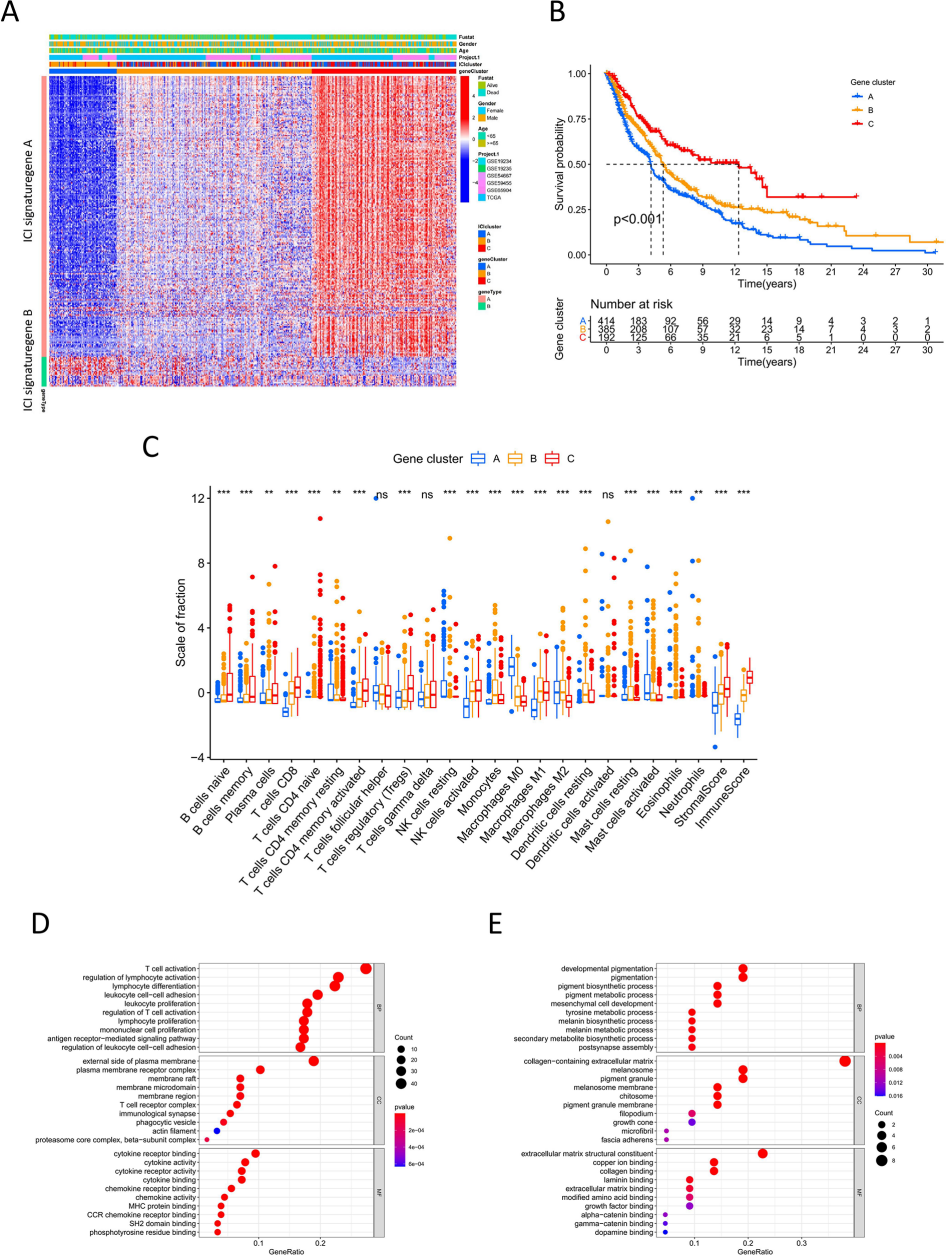
Immune gene subtype **(A)** Unsupervised clustering heatmap of differential expression genes among three ICI cluster. **(B)** Kaplan-Meier curves of overall survival for gene cluster A-C. Log rank test P <0.001. **(C)** The box plot of immune cells fraction in gene cluster A-C. **P < 0.01; ***P<0.001; ns, no significance. **(D, E)** GO enrichment analysis for ICI signature gene A and B.

To identify the difference in gene expression between immune subtypes, we analyzed gene expression among the three ICI clusters using the R package “limma”. Unsupervised clustering analysis was performed for 266 DEGs. The consensus matrix and gene cluster heatmap indicated that three gene clusters were optimal patterns (**Figures S2A–D**). Thus, DEGs were grouped into gene clusters A, B, and C (**Figure 2A**). These clusters showed significant differences in overall survival. Cluster C had better prognosis, whereas cluster A showed poor outcomes (log-rank test, P<0.001; **Figure 2B**). There was also a significant difference in ICI among gene clusters A-C (**Figure 2C**). Cluster C had a high immune score and stromal score and showed high infiltration of anti-tumor immune cells, such as CD8 T cells, NK cells, B cells, and M1 macrophages. Cluster A was characterized by low immune score and stromal score, thus showing worse prognosis. With respect to the expression of immune activity-related signature genes and immune checkpoint signature genes, Cluster C showed obvious superiority among the three groups (P<0.0001, **Figures S2E, F**).

For DEGs based on immune subtypes, genes positively and negatively associated with the gene cluster were defined as ICI gene signature A and ICI gene signature B, respectively. Gene dimension reduction using the Boruta algorithm obtained 211 signature genes (**Table S4**). In GO functional enrichment analysis, ICI gene signature A was significantly enriched in immune-related functions, while ICI gene signature B was mainly associated with biosynthesis and metabolism (**Figures 2D, E**).

### Construction of ICI Score

**Figure 3:**
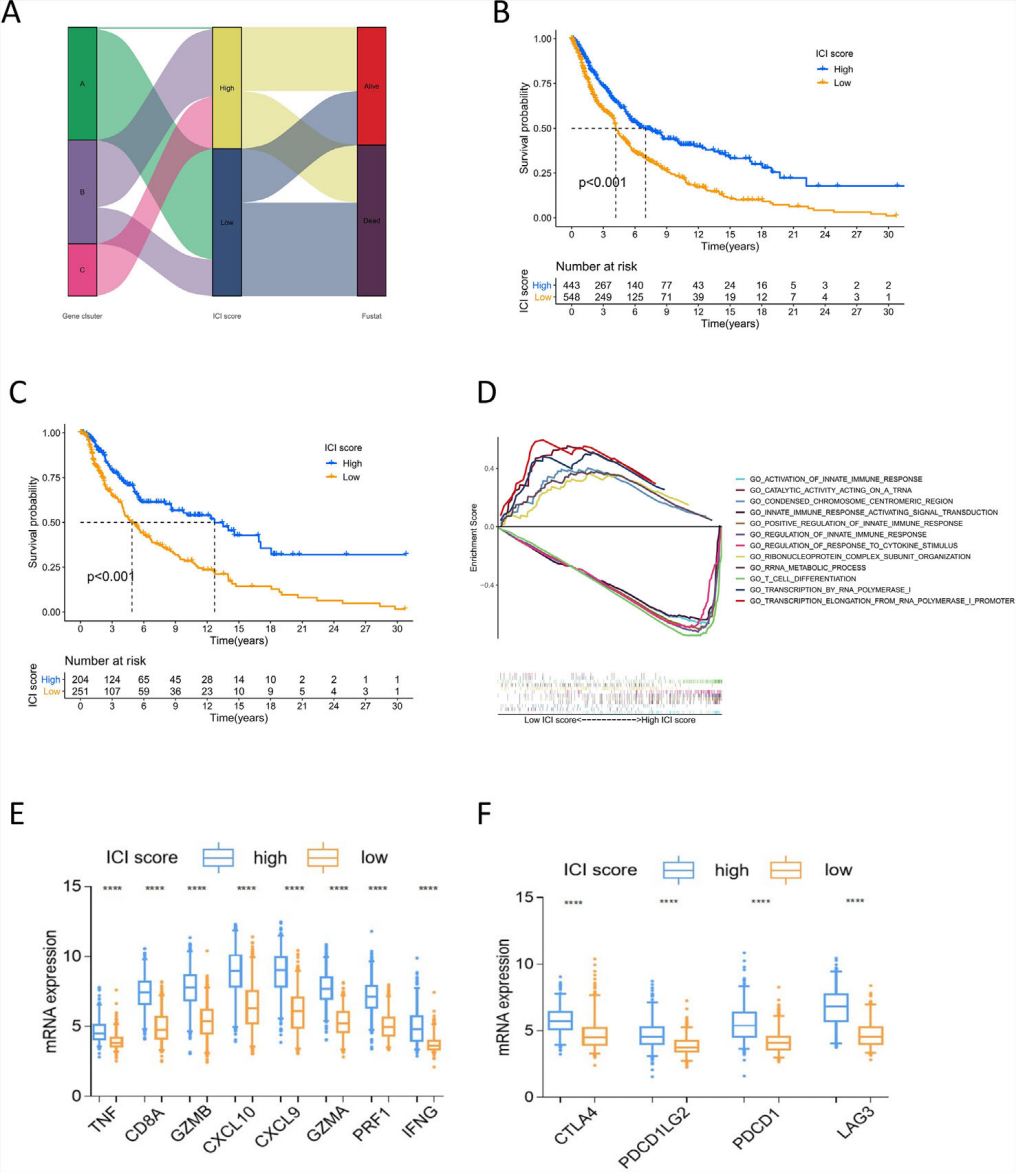
Analysis of ICI score. **(A)** Alluvial diagram of ICI scores groups distribution in different gene cluster, and survival outcomes. **(B)** Kaplan-Meier curves of overall survival for high and low ICI score cluster in all sample. Log rank test P <0.001. **(C)** Kaplan-Meier curves of overall survival for high and low ICI score cluster in TCGA cohort. Log rank test P <0.001. **(D)** GSEA of high and low ICI score groups for all melanoma samples. **(E)** The box plot of immune activity related signature genes expression (CXCL9, CXCL10, TNF, IFNG, CD8A, GZMA, GZMB, PRF1) in high and low ICI score, ****P<0.0001. **(F)** The box plot of immune checkpoint signature genes expression (CTLA4, PDCD1, PDCD1LG2, LAG3) in high and low ICI score, ****P<0.0001.

PCA was applied to calculate two aggregate scores: ICI scores A and B from ICI gene signature A and B. We computed ICI score A and ICI score B for each melanoma sample as the sum of the relevant individual scores. The prognostic signature score was then defined as the ICI score. All samples were grouped into the high or low ICI score group based on the optimal cutoff value (**Table S5**). The alluvial diagram displayed the distribution of the ICI score groups under different gene clusters and survival outcomes (**Figure 3A**). The low ICI score group was mostly composed of gene cluster A, which had a poor prognosis. Gene cluster C, related to better outcomes, was included in the high ICI score group. Survival analysis to identify the prognostic value of the ICI score showed that for all melanoma samples, the high-score group had superior overall survival over the low-score group (log-rank test, P<0.001; **Figure 3B**). Moreover, the prognostic value of the ICI score was validated in most datasets. The high ICI score group showed superior prognosis in the TCGA (log-rank test P<0.001, **Figure 3C**), GSE19234 (log-rank test P=0.049), GSE46517 (log-rank test P<0.001), GSE54467 (log-rank test P=0.033), and GSE65904 (log-rank test P=0.047) (**Figure S3**) datasets. These results confirm the value of the ICI score for prognostic prediction. GSEA showed that the high ICI score group had prominently enriched immune-related pathways, including innate immune response, positive regulation of innate immune response, T cell differentiation, and response to cytokine stimulus. Meanwhile, the low ICI score group mainly showed enriched biosynthesis (**Figure 3D**). The high ICI score group showed significant immune activity, with higher expression of immune activity signature genes and immune checkpoint signature genes compared with that of the low ICI score group (**Figures 3E, F**).

### ICI Score and Cancer Somatic Variants

**Figure 4:**
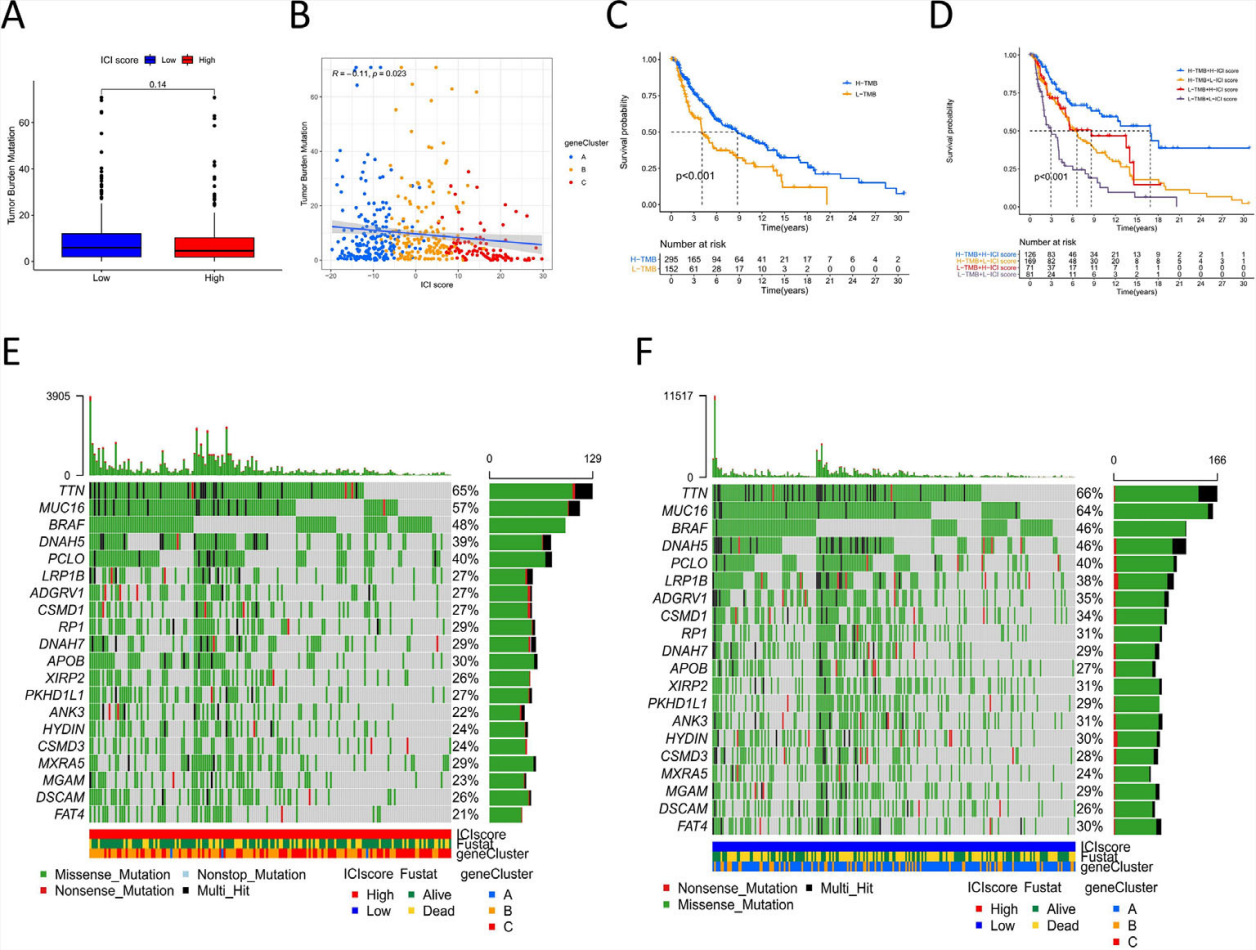
The correlation between ICI score and cancer somatic variants. **(A)** TMB difference among the high and low ICI score groups. **(B)** The correlation Scatter plots between TMB and ICI score. **(C)** Kaplan-Meier curves of overall survival for high and low TMB group. Log rank test P <0.001. **(D)** Kaplan-Meier curves of overall survival stratified by both TMB and ICI scores. Log rank test P <0.001. **(E, F)** The oncoPrint of high (left) and low (right)ICI score. Individual patients represented in each column. Missense mutation: green; Nostop mutation: gray; Nonsense mutation: red; Multi-hit: black. The top bar plot represented TMB. The right bar plot shows the mutation frequency of each gene in separate ICI score groups. The below bar represented ICI cluster, gene cluster and survival outcome.

Tumor genomic mutations lead to the occurrence of neoantigens, which are favorable for immune therapy (22). TMB is a predictive factor for treatment response to immune therapy (23). To investigate the intrinsic correlation between TMB and ICI scores, we downloaded the data of melanoma somatic variants from the TCGA database. We compared the TMB between melanoma samples with high ICI scores and those with low ICI scores. The box plot showed a difference in mutation frequency between the high and low ICI score groups, but the difference was not significant (**Figure 4A**). Correlation analyses showed that ICI scores were negatively correlated with TMB (Spearman coefficient: R=-0.11, P=0.023, **Figure 4B**). High TMB patients had better survival outcomes than did low TMB patients (log-rank test P<0.001, **Figure 4C**). We then evaluated the synergistic effect of ICI scores on prognostic stratification. Stratified survival analysis showed significant differences in survival according to the ICI scores among the TMB subgroups (log-rank P<0.001, **Figure 4D**). These results indicate that the ICI score could be a predictor of survival independent of the TMB.

The distribution of melanoma somatic variants between the low and high ICI score groups was accessed by R package “maftools.” The top 20 driver genes with the highest alteration frequency are shown in **Figures 4E, F**. The alteration frequencies of FAT4, LRP1B, and ANK3 were significantly different between the low and high ICI score groups (**Table 1**).

**Table 1:** The Correlation between the ICI Scores and Somatic Variants.

Gene	H-wild	H-mutation	L-wild	L-mutation	P value
GLRB	192(97.46%)	5(2.54%)	221(88.4%)	29(11.6%)	0.000653446
ACSM1	192(97.46%)	5(2.54%)	226(90.4%)	24(9.6%)	0.004861082
ITGAX	194(98.48%)	3(1.52%)	231(92.4%)	19(7.6%)	0.006359613
SEL1L3	193(97.97%)	4(2.03%)	229(91.6%)	21(8.4%)	0.006885403
LRRC4C	185(93.91%)	12(6.09%)	214(85.6%)	36(14.4%)	0.007742623
CATSPERD	178(90.36%)	19(9.64%)	242(96.8%)	8(3.2%)	0.008300153
MYH4	172(87.31%)	25(12.69%)	193(77.2%)	57(22.8%)	0.008825349
PKHD1	183(92.89%)	14(7.11%)	211(84.4%)	39(15.6%)	0.009043977
ATP13A4	188(95.43%)	9(4.57%)	220(88%)	30(12%)	0.009448039
IGSF10	184(93.4%)	13(6.6%)	213(85.2%)	37(14.8%)	0.009879668
EXPH5	189(95.94%)	8(4.06%)	222(88.8%)	28(11.2%)	0.0099167
RXFP2	193(97.97%)	4(2.03%)	230(92%)	20(8%)	0.010213103
PDGFRA	192(97.46%)	5(2.54%)	228(91.2%)	22(8.8%)	0.010494566
MPP7	187(94.92%)	10(5.08%)	219(87.6%)	31(12.4%)	0.012475552
FHDC1	193(97.97%)	4(2.03%)	231(92.4%)	19(7.6%)	0.015072759
BTBD11	191(96.95%)	6(3.05%)	227(90.8%)	23(9.2%)	0.015127514
ADGRF4	192(97.46%)	5(2.54%)	229(91.6%)	21(8.4%)	0.015293351
CSF2RB	192(97.46%)	5(2.54%)	229(91.6%)	21(8.4%)	0.015293351
TGM6	182(92.39%)	15(7.61%)	244(97.6%)	6(2.4%)	0.018201576
LRP1B	143(72.59%)	54(27.41%)	154(61.6%)	96(38.4%)	0.019185781
SZT2	190(96.45%)	7(3.55%)	226(90.4%)	24(9.6%)	0.020842861
DNAH1	173(87.82%)	24(12.18%)	236(94.4%)	14(5.6%)	0.021072363
IGHM	192(97.46%)	5(2.54%)	230(92%)	20(8%)	0.022153014
PPP1R3A	176(89.34%)	21(10.66%)	203(81.2%)	47(18.8%)	0.02467418
TRPC4	184(93.4%)	13(6.6%)	216(86.4%)	34(13.6%)	0.02506211
ZNF99	184(93.4%)	13(6.6%)	216(86.4%)	34(13.6%)	0.02506211
NBEA	182(92.39%)	15(7.61%)	213(85.2%)	37(14.8%)	0.027528675
PDZD2	185(93.91%)	12(6.09%)	218(87.2%)	32(12.8%)	0.027530981
PCDHB7	189(95.94%)	8(4.06%)	225(90%)	25(10%)	0.027672056
FAT4	156(79.19%)	41(20.81%)	174(69.6%)	76(30.4%)	0.029178661
PLEKHH2	192(97.46%)	5(2.54%)	231(92.4%)	19(7.6%)	0.031882714
GRM4	193(97.97%)	4(2.03%)	233(93.2%)	17(6.8%)	0.032281736
CACNA2D4	193(97.97%)	4(2.03%)	233(93.2%)	17(6.8%)	0.032281736
PTPRB	174(88.32%)	23(11.68%)	201(80.4%)	49(19.6%)	0.032897855
FCGBP	182(92.39%)	15(7.61%)	214(85.6%)	36(14.4%)	0.03656706
SI	175(88.83%)	22(11.17%)	203(81.2%)	47(18.8%)	0.037014862
FLG	158(80.2%)	39(19.8%)	178(71.2%)	72(28.8%)	0.037794192
PCDHA12	189(95.94%)	8(4.06%)	226(90.4%)	24(9.6%)	0.038406702
HTT	189(95.94%)	8(4.06%)	226(90.4%)	24(9.6%)	0.038406702
SERPINB7	180(91.37%)	17(8.63%)	241(96.4%)	9(3.6%)	0.040168226
ACE	190(96.45%)	7(3.55%)	228(91.2%)	22(8.8%)	0.041099422
TRPM6	181(91.88%)	16(8.12%)	213(85.2%)	37(14.8%)	0.043279955
EML5	191(96.95%)	6(3.05%)	230(92%)	20(8%)	0.043557638
PRLR	187(94.92%)	10(5.08%)	223(89.2%)	27(10.8%)	0.044684979
SLITRK3	187(94.92%)	10(5.08%)	223(89.2%)	27(10.8%)	0.044684979
FSIP2	184(93.4%)	13(6.6%)	218(87.2%)	32(12.8%)	0.044972848
PLCE1	179(90.86%)	18(9.14%)	210(84%)	40(16%)	0.045282963
ANK3	153(77.66%)	44(22.34%)	172(68.8%)	78(31.2%)	0.047485414
DNAH10	169(85.79%)	28(14.21%)	195(78%)	55(22%)	0.047763319
DUOX2	188(95.43%)	9(4.57%)	225(90%)	25(10%)	0.048733515

### Predictive Value of the ICI Scores for Clinical Characteristics and Immunotherapy Benefit

**Figure 5:**
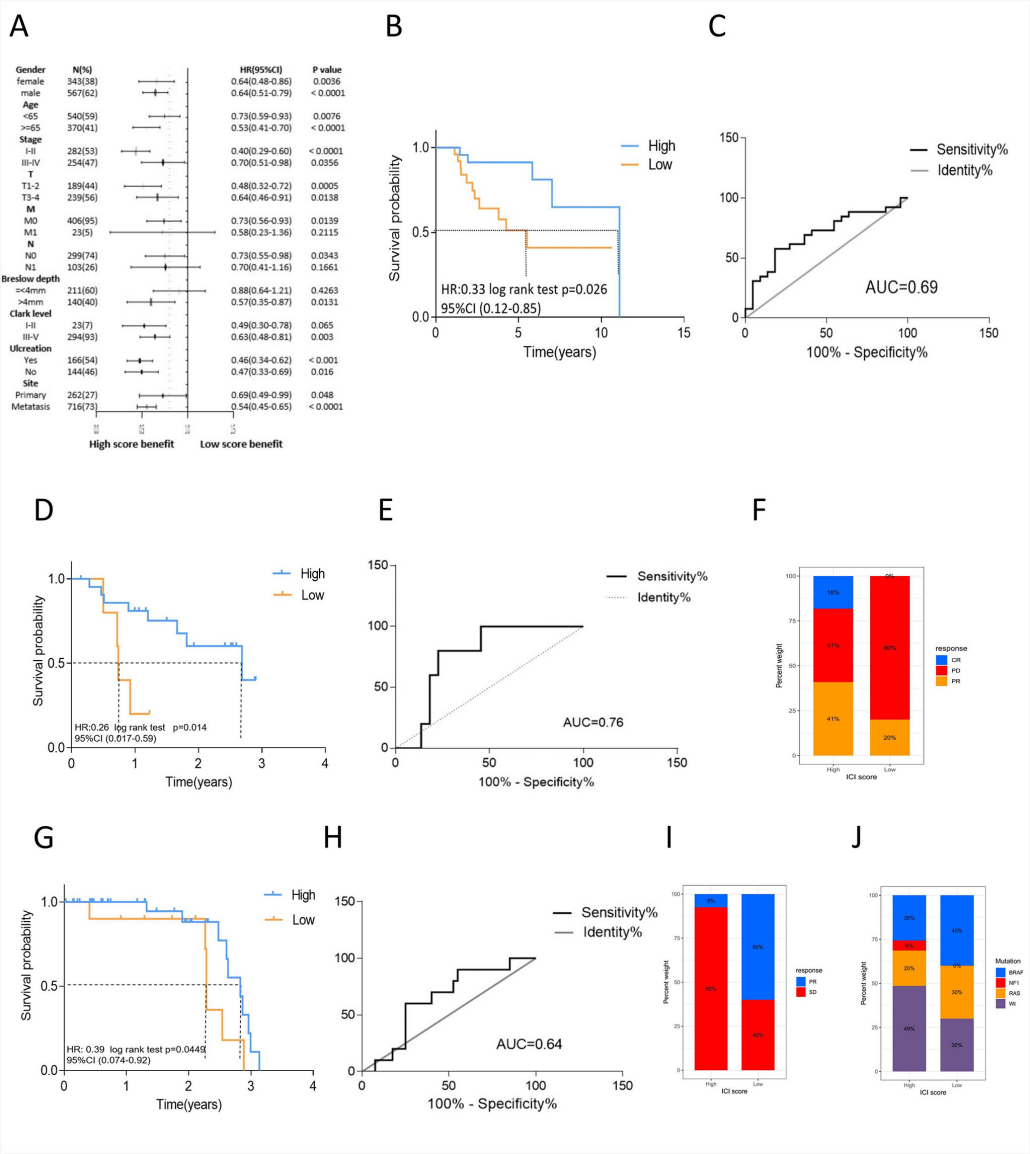
The role of ICI scores in the evaluation of melanoma clinical characteristics and immune therapy benefit. **(A)** The forest plot of stratified survival analysis for clinical indicator based on ICI score. The length of the horizontal line represents the 95% CI for each group, the sample number, HR and 95%CI as well as P value of each group were listed. **(B)** Kaplan-Meier curves of overall survival for high and low ICI score cluster in GSE19423 cohort. Log rank test P =0.026. **(C)** The predictive value of the ICI score measured by ROC curves in GSE19423. **(D)** Kaplan-Meier curves of overall survival for high and low ICI score cluster in GSE78220. Log rank test P =0.014. **(E)** The predictive value of the ICI score measured by ROC curves in GSE78220. **(F)** Rate of clinical response to anti-PD1 treatment in high and low ICI score groups in GSE78220. **(G)** Kaplan-Meier curves of overall survival for high and low ICI score cluster in CA209038 cohort. Log rank test P =0.0449. **(H)** The predictive value of the ICI score measured by ROC curves in CA209038. **(I)** Rate of clinical response to anti-PD1 treatment in high and low ICI score groups in CA209038 cohort. **(J)** Mutation frequency between high and low ICI score group in CA209038 cohort.

We then assessed the predictive value of the ICI score for the clinical characteristics and immune therapy benefit in melanoma. Combining the ICI score and clinical information, we analyzed the main clinical indicators, including age, sex, TMN stage, Breslow depth, Clark level, ulceration and tumor site. A forest plot was created for the stratified survival analysis based on ICI score, and significant differences were observed (**Figure 5A**). In all factors, except for M1, N1, and <4 mm Breslow depth, those with high scores had significantly superior overall survival to those with low scores. Breslow depth, Clark level, and ulceration are specific factors for melanoma (23). In the metastatic melanoma GSE22154 cohort, the high ICI score group had a higher survival (**Figure S4G**). Our results support that the ICI score could predict survival independent of these clinical indicators.

For the prognostic value of the ICI score with respect to the immune therapy benefit, three cohorts were evaluated. In the GSE19423 cohort, the high ICI score group had a higher overall survival after immune therapy (log rank test P=0.026, **Figures 5B, C**). In the GSE78220 cohort, the high ICI score group had better survival outcomes than did the low score group (log rank P=0.014, **Figures 5D, E**). The partial response rate to anti-PD-L1 therapy was also higher in the high ICI score group than that in the low score group. Further, the rate of progressive disease was significantly lower in the high ICI score group. Notably, some patients in the high ICI score group exhibited complete response, while none of the patients in the low score group achieved complete response (**Figure 5F**). The CA209038 cohort showed similar results. The high ICI score group had a greater survival benefit from immune therapy (log rank P=0.0449, **Figures 5G, H**) and a higher response rate to therapy (**Figure 5I**). The low ICI score group exhibited higher mutation frequency, consistent with our previous result (**Figure 5J**).

We compared the predictive ability of the signature genes in our study and other models in terms of the immunotherapy benefit. We selected signature genes identified by the INF model and the HNSC model (15, 24). The Kaplan-Meier curves and the ROC curves of the ICI score of the other two models are shown in Fig S4A-F. For the other two models, there were no statistically significant differences in the overall survival between those with high and low ICI scores, and the prognosis differed among the cohorts. The ROC curve showed that the predictive value was low. The result indicated that the signature genes that we used had better predictive value and robustness in terms of clinical outcomes among melanoma patients.

## Discussion

Melanoma is one of the most immunogenic tumors because of its high genomic mutational burden, thus its great potential to respond favorably to immune therapy (25). Immune checkpoint blockade therapy, including anti-PD1, anti-PDL1, and anti-CTLA-4, has been proven to improve the overall survival of patients with advanced melanoma (26, 27). PD-L1 and TMB have been shown to be independent, not correlated, predictive variables of the benefit of immune therapy (23). However, no single factor has been validated to adequately predict the treatment benefit of immune therapy (28). The interaction between tumor cells and the microenvironment leads to a tumor-driven immune response (9). The increasing evidence on the role of TILs in primary and metastatic melanomas supports that the immune system is involved in the progression and outcomes of melanoma (29, 30). Thus, algorithms for identifying melanoma prognosis and treatment benefit based on qualitative and quantitative characteristics of tumor and immune cells are expected (31). In this study, we analyzed the melanoma immune landscape and constructed an ICI score based on the immune microenvironment to evaluate prognosis and immune therapy benefit.

Three major immune phenotypes have been proposed in cancer, namely the immune-inflamed, immune-excluded, and immune-desert phenotypes (10, 32). In our study, ICI cluster A exhibited the immune-inflamed phenotype which is characterized by the presence of CD4 and CD8 T cells in the tumor parenchyma (33). ICI cluster B exhibited the immune-desert phenotype, which is characterized by an absence of ICI in the tumor parenchyma and stroma. This phenotype is considered to be a non-inflamed tumor (34). ICI cluster C exhibited the immune-excluded phenotype, with a high level of infiltration by B cells, naive and conventional T cells, Treg cells, and dendritic cells, as well as the highest stromal score. However, another study found that many immune cells only infiltrated the tumor margins due to the paucity of tumor stroma (35). ICI cluster C was similar to tertiary lymphoid structures (TLSs) which were present along the invasive tumor margin and stroma, and worked as sites of immune cell recruitment and activation (10). This may explain why ICI cluster C had a poorer prognosis than ICI cluster A. The results showed that the immune-inflamed phenotype had better overall survival, which is consistent with previous research which has shown that the presence of high levels of ICI is associated with favorable outcomes (29). We then analyzed the DEGs from the ICI cluster and defined three gene clusters related to immune activity. Similar to ICI clusters, gene clusters function better in distinguishing immune activity and survival outcomes. Gene clusters related with longer overall survival showed activated immune function, whereas gene clusters related to poor outcome had low levels of anti-tumor immune cells and low expression of immune signature genes. These findings on the immune landscape of melanoma warrant further studies considering the lack of independent biomarkers in this malignancy.

Some researchers have proposed the phenotype of T cell infiltration as a predictor of response to immune therapy (36). Considering the correlation between the immune subtypes of melanoma and survival, we constructed ICI scores to evaluate survival prognosis, clinical characteristics, and immune therapy benefit. The ICI score was defined by immune signature genes from gene cluster results. The high ICI score group showed prominent enrichment of immune-related pathways and high expression of immune signature genes, exhibiting a “hot” tumor phenotype. In addition, the immune phenotype was associated with better prognosis. Somatic mutations in tumors lead to neoantigens that are recognized and targeted by the immune system (22). It has been reported that low TMB conferred poor survival outcomes in melanoma and was associated with lower age and advanced pathological stage (5). Similarly, our results showed that high TMB conferred a survival benefit. Interestingly, we found that the ICI score was negatively correlated to TMB. A previous study found that a higher mutation frequency was not indicative of a higher level of ICI (37). Thus, a high TMB does not necessarily result in a high ICI score. Our stratified survival analysis showed that the ICI score could be used to evaluate prognosis. Particularly, both ICI score and TMB were independent predictors of outcome.

TMB and PD1 have been reported as predictive variables of the benefit of immune therapy (23). In our research, the ICI score was an effective factor to predict the benefit of immune therapy for melanoma patients. A clinical trial reported that the presence of CD8+ T cells was favorable for DFS in immune therapy (38). In our study, the high ICI score group benefited more from immune therapy and exhibited activated immune status than did the low ICI score group. This demonstrated that the effect of immune therapy relied on the tumor immune microenvironment. The malignant phenotypes of cancers are determined not only by the intrinsic properties of cancer cells, but also by components of the TME (39). In melanoma, tumor stage, tissue site, ulceration, thickness, and patient age and sex are associated with immune infiltration (40). Our result is in accordance with those of a previous study. Further, stratified survival analysis showed that in all primary clinical characteristics, those with high ICI scores had superior prognosis to those with low scores. In the Clark level, Breslow depth, and ulceration, three tumor-specific factors, the ICI score also robustly predicted the clinical benefit of immune therapy (41, 42). Collectively, these findings support that the ICI score is an independent prognostic biomarker. There are some limitations to the study. As the clinical information in the datasets had limited information on surgery and radiation therapy, we were unable to include these two clinical characteristics in the assessment of the predictive value of the ICI score. More clinical data was needed to support the current conclusions.

In summary, we analyzed the immune microenvironment landscape and developed an ICI score for predicting survival and immune therapy benefit in patients with melanoma, by assessing the immune microenvironment. Our results will facilitate understanding of the melanoma tumor microenvironment and provide a new immune therapy strategy.

## Data Availability Statement

The datasets presented in this study can be found in online repositories. The names of the repository/repositories and accession number(s) can be found in the article/**Supplementary Material**.

## Author Contributions

Concept and Design: DL. Data collection, Analysis: DL, XY. Drafting: DL, XY. Revising: DL, XY. Supervision for the study: XW. All authors contributed to the article and approved the submitted version.

## Funding

This work was supported by the National Natural Science Foundation of China (81473441).

## Conflict of Interest

The authors declare that the research was conducted in the absence of any commercial or financial relationships that could be construed as a potential conflict of interest.

## Supplementary Material

Supplementary Figure 1
**(A)** Overview of study design. **(B–E)** Consensus matrixes (K=3-6) of ICI cluster for all melanoma sample. **(F)** Unsupervised clustering heatmap of immune cells infiltration for TCGA cohort. Rows represent tumor-infiltrating immune cells, and columns represent samples.

Supplementary Figure 2
**(A–D)** Consensus matrixes (K=3-6) of gene cluster for all melanoma sample. **(E)** The box plot of immune activity related signature genes expression (CXCL9, CXCL10, TNF, IFNG, CD8A, GZMA, GZMB, PRF1) between gene cluster A-C, ****P<0.0001. **(F)** The box plot of immune checkpoint signature genes expression (CTLA4, PDCD1, PDCD1LG2, LAG3) between gene cluster A-C, ****P<0.0001.

Supplementary Figure 3 Kaplan-Meier curves of overall survival for high and low ICI score cluster in each datasets. **(A)**, GSE19234; **(B)**, GSE45617; **(C)**, GSE54467; **(D)**, GSE59455; **(E)**, GSE69504.

Supplementary Figure 4 Systematic Comparision. **(A)** Kaplan-Meier curve and ROC curve of INF model in GSE19423 cohort. **(B)** Kaplan-Meier curve and ROC curve of INF model in GSE78220 cohort. **(C)** Kaplan-Meier curve and ROC curve of INF model in CA209038 cohort. **(D)** Kaplan-Meier curve and ROC curve of HNSC model in GSE19423 cohort. **(E)** Kaplan-Meier curve and ROC curve of HNSC model in GSE78220 cohort. **(F)** Kaplan-Meier curve and ROC curve of HNSC model in CA209038 cohort. **(G)** Kaplan-Meier curve and ROC curve of ICI score in GSE22154 cohort.

Supplementary Figure 5 The box plot of immune cells fraction in ICI cluster A-C estimated by xCell Algorithm.
