# Supplementary figures and images for: Tumor Immune Microenvironment Characterization Identifies Prognosis and Immunotherapy-Related Gene Signatures in Melanoma
Source: Front Immunol. 2021 May 6;12:663495. doi: 10.3389/fimmu.2021.663495 (PMC8134682; doi:10.3389/fimmu.2021.663495)

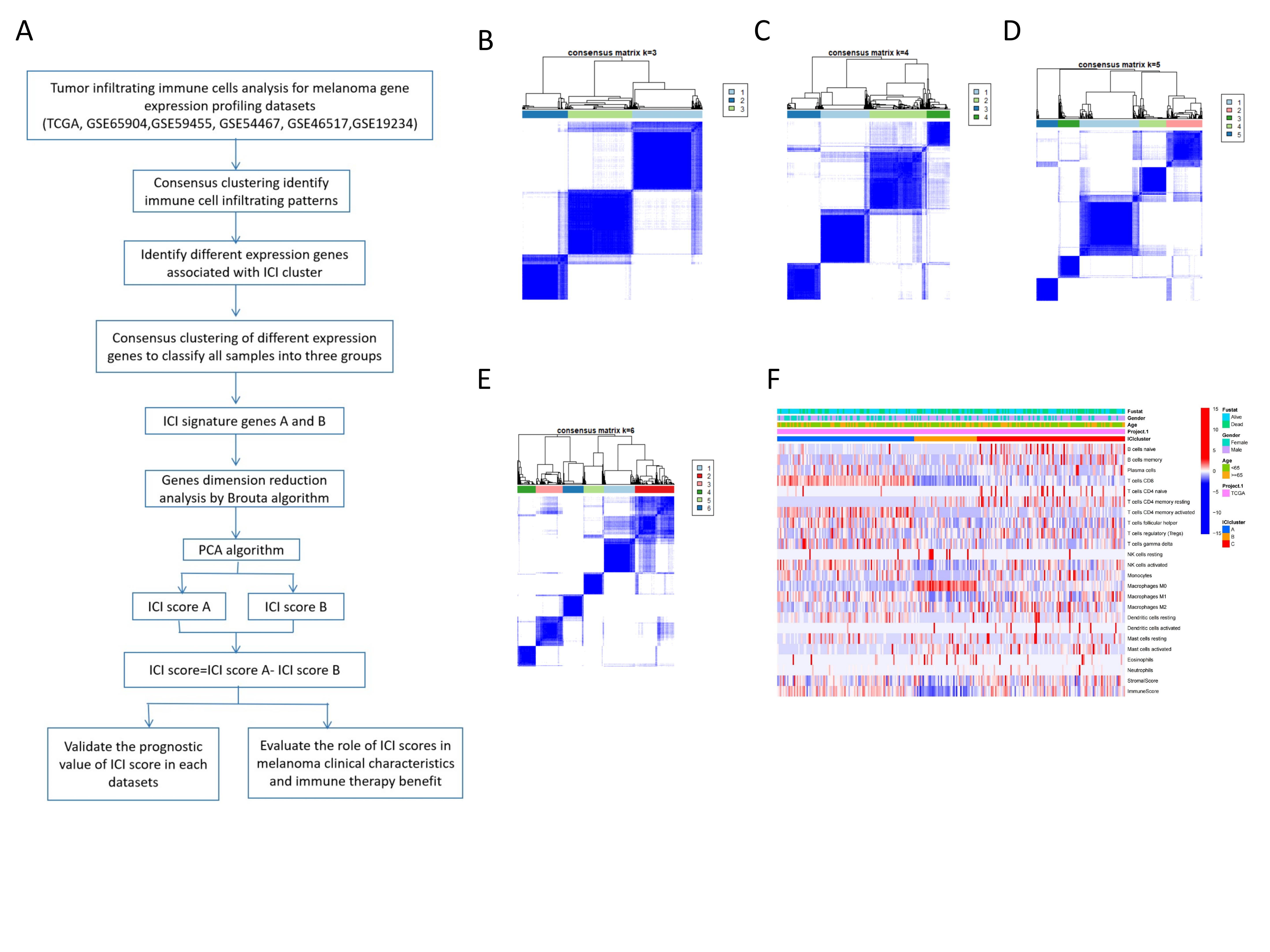

Supplement: Supplementary Figure 1 — (A) Overview of study design. (B–E) Consensus matrixes (K=3-6) of ICI cluster for all melanoma sample. (F) Unsupervised clustering heatmap of immune cells infiltration for TCGA cohort. Rows represent tumor-infiltrating immune cells, and columns represent samples. [file Image_1.jpeg]

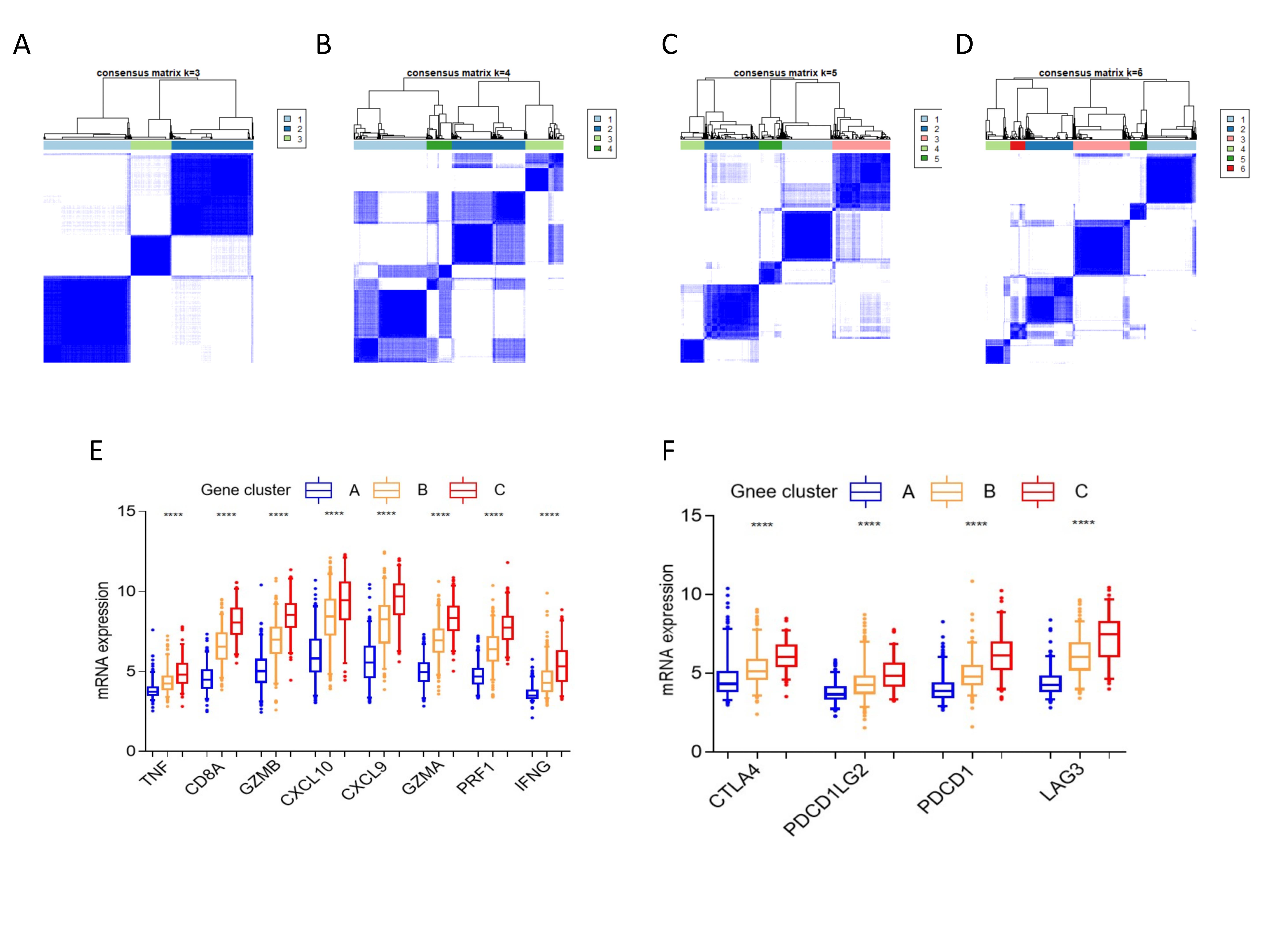

Supplement: Supplementary Figure 2 — (A–D) Consensus matrixes (K=3-6) of gene cluster for all melanoma sample. (E) The box plot of immune activity related signature genes expression (CXCL9, CXCL10, TNF, IFNG, CD8A, GZMA, GZMB, PRF1) between gene cluster A-C, ****P<0.0001. (F) The box plot of immune checkpoint signature genes expression (CTLA4, PDCD1, PDCD1LG2, LAG3) between gene cluster A-C, ****P<0.0001. [file Image_2.jpeg]

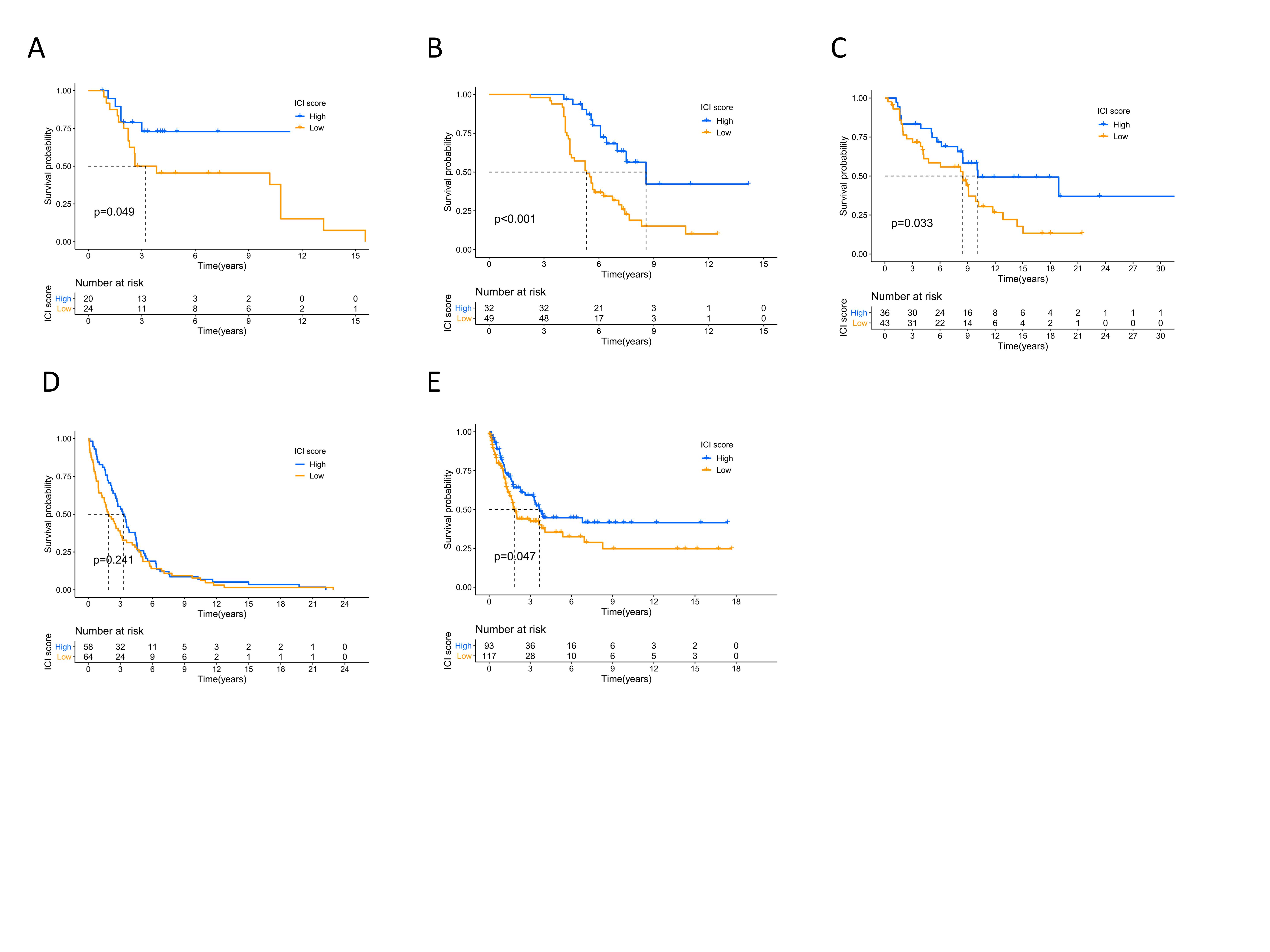

Supplement: Supplementary Figure 3 — Kaplan-Meier curves of overall survival for high and low ICI score cluster in each datasets. (A), GSE19234; (B), GSE45617; (C), GSE54467; (D), GSE59455; (E), GSE69504. [file Image_3.jpeg]

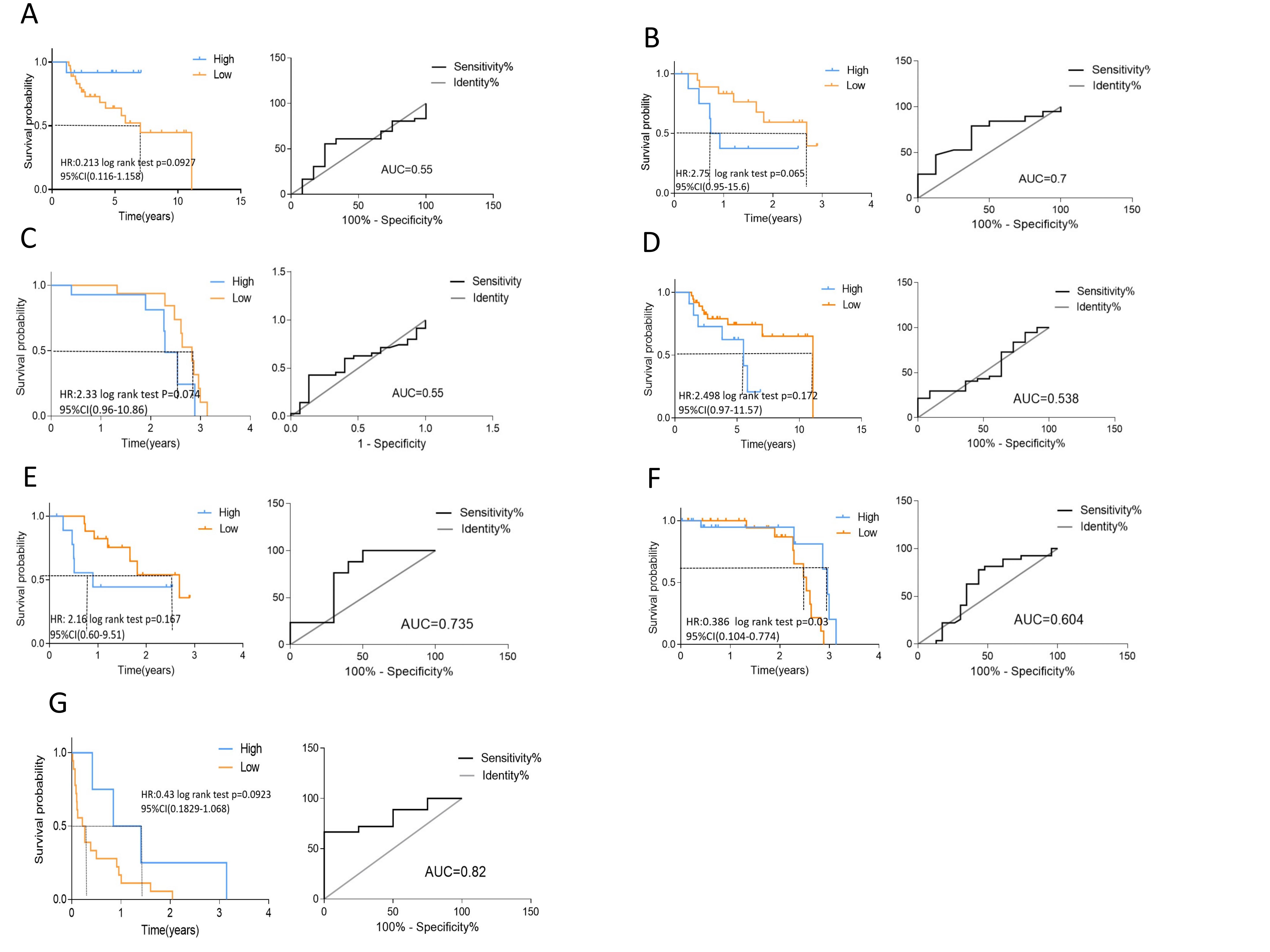

Supplement: Supplementary Figure 4 — Systematic Comparision. (A) Kaplan-Meier curve and ROC curve of INF model in GSE19423 cohort. (B) Kaplan-Meier curve and ROC curve of INF model in GSE78220 cohort. (C) Kaplan-Meier curve and ROC curve of INF model in CA209038 cohort. (D) Kaplan-Meier curve and ROC curve of HNSC model in GSE19423 cohort. (E) Kaplan-Meier curve and ROC curve of HNSC model in GSE78220 cohort. (F) Kaplan-Meier curve and ROC curve of HNSC model in CA209038 cohort. (G) Kaplan-Meier curve and ROC curve of ICI score in GSE22154 cohort. [file Image_4.jpeg]

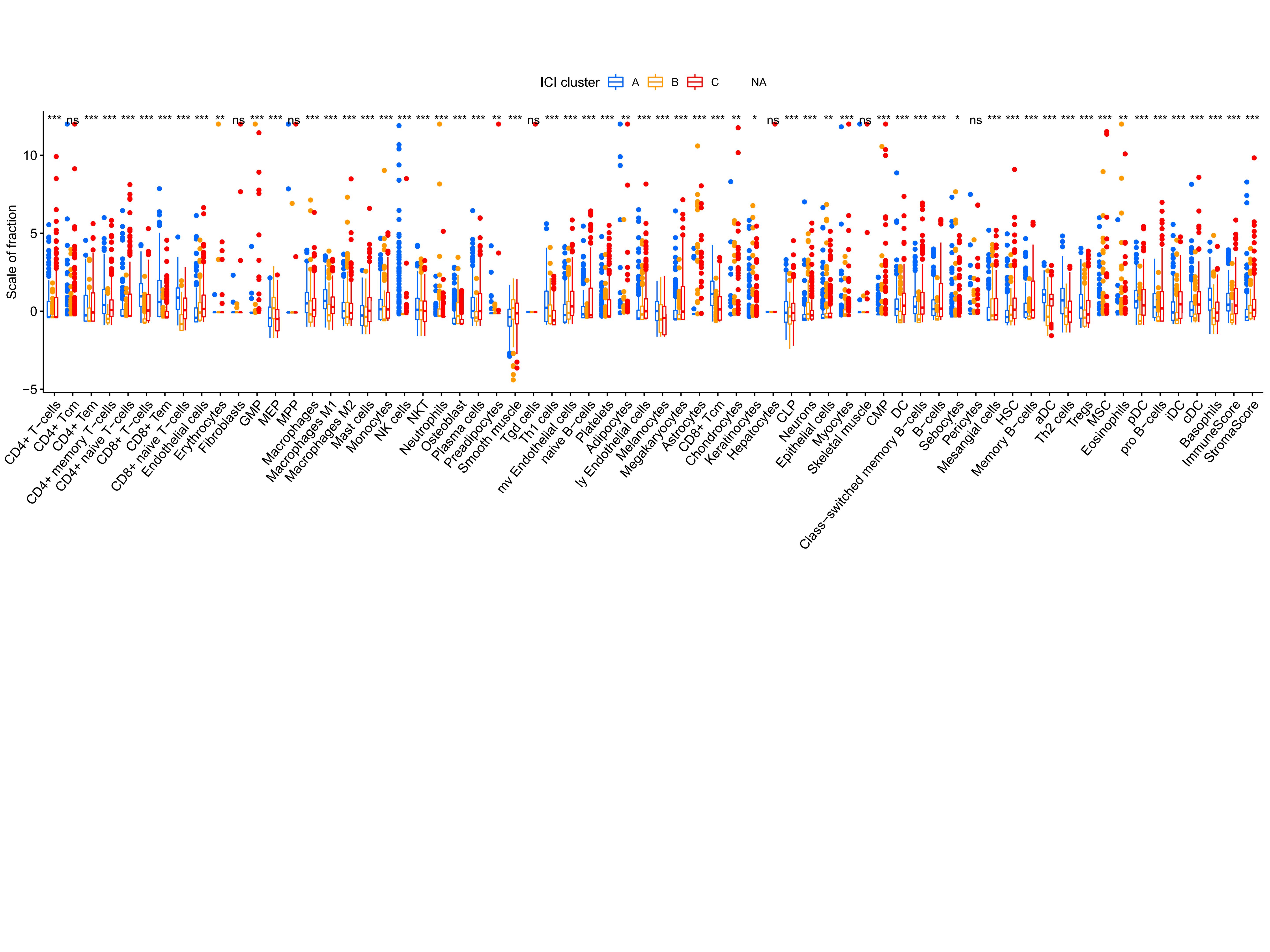

Supplement: Supplementary Figure 5 — The box plot of immune cells fraction in ICI cluster A-C estimated by xCell Algorithm. [file Image_5.jpeg]
